# 24-Epibrassinolide modulates antioxidant regulation and redox homeostasis in soybean exposed to cadmium stress

**DOI:** 10.1007/s12298-026-01754-y

**Published:** 2026-04-27

**Authors:** Furkan Demirel, Yonca Surgun-Acar

**Affiliations:** 1 School of Graduate Studies, Çanakkale Onsekiz Mart University, Çanakkale, 17000 Turkey; 2 Department of Agricultural Biotechnology, Faculty of Agriculture, Çanakkale Onsekiz Mart University, Çanakkale, 17000 Turkey

**Keywords:** *Glycine max*, Cadmium, 24-Epibrassinolide, Antioxidant system, Redox homeostasis, Gene expression

## Abstract

Cadmium (Cd) is a toxic heavy metal that severely impairs plant growth and productivity by disrupting cellular homeostasis. This study investigated the effects of foliar-applied 24-Epibrassinolide (EBL) on antioxidant capacity and redox coordination in Cd-stressed *Glycine max* (L.) through integrated physiological, biochemical, and transcriptional analyses. Seedlings were grown hydroponically and exposed to 20 or 40 mg L^− 1^ Cd for 10 days, while 1 µM EBL was applied as a foliar treatment. Increasing Cd concentrations reduced plant growth and photosynthetic pigment contents, whereas Cd accumulation in leaves increased markedly. The combined application of Cd and EBL significantly improved shoot growth and pigment contents compared to plants treated with Cd alone. Cadmium stress increased hydrogen peroxide and malondialdehyde levels, while co-application of EBL significantly decreased these oxidative markers. Exposure to Cd stimulated the activities of key antioxidant enzymes, including superoxide dismutase, catalase, ascorbate peroxidase, and peroxidase, with EBL application further enhancing these activities, particularly at 20 mg L^− 1^ Cd. In addition, EBL promoted the accumulation of non-enzymatic antioxidants, including proline, phenolics, and flavonoids. Transcriptional analysis revealed that EBL coordinately regulates antioxidant-related gene expression, supporting a gene-specific response under Cd stress. Overall, foliar application of EBL strengthened antioxidant defences and maintained redox homeostasis, thereby improving soybean tolerance, particularly at the lower Cd concentration.

**Supplementary Information:**

The online version contains supplementary material available at 10.1007/s12298-026-01754-y.

## Introduction

Heavy metal pollution in soil has increased at an alarming rate, threatening agricultural productivity and ecosystem sustainability. Among heavy metals, cadmium (Cd) in its^+^2-oxidation state is highly reactive, non-essential, and persistent in the environment, causing severe hazards to both animals and plants even at low concentrations (Shanmugaraj et al. 2019; Qin et al. 2020). In plants, Cd is primarily absorbed by roots via membrane transporters that normally facilitate the uptake of essential mineral nutrients, including calcium, zinc, and iron (Riaz et al. 2021). Cadmium exposure triggers a range of detrimental effects in plants, which collectively inhibit plant growth and ultimately lead to substantial yield losses (Chen et al. 2025). To counteract Cd toxicity, plants employ multiple regulatory mechanisms, including reinforcement of antioxidant defences, compartmentalization of Cd within specific cellular or tissue regions, chelation through metallothioneins and phytochelatins, and active sequestration or efflux of the metal (Shahid et al. 2017; Rizwan et al. 2018).

The exogenous application of phytohormones has been widely reported as an effective strategy to alleviate the toxic effects of heavy metals in plants (Sytar et al. 2019). Brassinosteroids (BRs) are naturally occurring, biodegradable polyhydroxylated steroids that play central regulatory roles in plant growth, development, and stress adaptation (Li et al. 2021; Chakraborty et al. 2025). Increasing evidenc suggests that BRs enhances plant tolerance to abiotic stresses through interconnected physiological and biochemical mechanisms. These include maintaining photosynthetic efficiency, stimulating antioxidant enzyme activities, promoting osmoprotectant accumulation, and modulating endogenous phytohormone biosynthesis and signaling pathways, such as gibberellins (GA) and abscisic acid (ABA) (Yang et al. 2023). Exogenous 24-Epibrassinolide (EBL), an active BR, has similarly been shown to mitigate metal-induced toxicity in several plant species, including nickel stress in *Raphanus sativus* (Sharma et al. 2011), copper stress in *Vitis vinifera*, chromium stress in *Capsicum annuum* (Mumtaz et al. 2022), lead stress in *Lycopersicon esculentum* (Maia et al. 2022), and arsenate stress in *Oryza sativa* (Shabab and Sarada 2024).

Soybean (*Glycine max* L.) is one of the five most important food crops cultivated worldwide and represents the most widely grown oilseed crop (Kumari et al. 2025). The adverse effects of Cd on soybean growth and physiology have been widely documented in previous studies (Balestrasse et al. 2001; Ferreira et al. 2006; Finger-Teixeira et al. 2010; Chmielowska-Bąk et al. 2013; Xue et al. 2013; Pérez Chaca et al. 2014; Ikhajiagbe et al. 2021; Liu et al. 2023). Nevertheless, the role of BRs in regulating antioxidant defence mechanisms in soybean under Cd stress remains unclear. To address this gap, we investigated how exogenous EBL influences enzymatic and transcriptional responses under oxidative stress induced by varying Cd concentrations. Within this framework, the effects of foliar-applied EBL on growth, oxidative stress markers, activities of key antioxidant enzymes, and stress-related gene expression were evaluated in Cd-stressed soybean seedlings.

## Materials and methods

### Plant material and experimental design

Soybean [*Glycine max* (L.) Merr. cv. Lider] seeds were obtained from the ProGen Seed Company (Hatay, Türkiye). The cultivar Lider was selected as a well-characterized variety that has been widely used in agronomic and physiological studies under diverse environmental conditions (Yaşar and Sezgin 2022; Yılmaz 2024; Ingkar et al. 2025). Cadmium chloride (CdCl_2_) concentrations used in this study were determined through a preliminary experiment. Soybean seeds were surface-sterilized following Aserse et al. (2012) and incubated for five days in Petri dishes on filter papers moistened with 0, 5, 10, 20, 40, and 80 mg L^− 1^ CdCl_2_. Based on root length measurements, CdCl_2_ concentrations of 20 mg and 40 mg L^− 1^ (EC₅₀) were chosen. For the subsequent experiments, soybean seeds were surface-sterilized and germinated using the same protocol as in the preliminary experiment, then transferred to a hydroponic culture system containing one-quarter-strength (¼) Hoagland nutrient solution (Hoagland and Arnon 1938). Plants were maintained in a controlled growth chamber at 22 ± 2 °C, under a 16/8 h light/dark photoperiod with a light intensity of 225 µmol m^− 2^ s^− 1^. Seedlings were acclimated to hydroponic conditions for three days prior to treatment. Then, plants were exposed to ¼-strength Hoagland solution supplemented with 20 or 40 mg L^− 1^ CdCl_2_ for 10 days. Foliar application of 1 µM EBL was carried out following the method of Surgun et al. (2016), using a spray volume of approximately 1 ml per plant, and repeated every two days. Nutrient solutions were renewed every two days throughout the experimental period. Each treatment was conducted using three independent glass hydroponic tanks. For biochemical analyses, four independent biological replicates were used per treatment, whereas quantitative real-time PCR (qRT-PCR) was performed with three biological replicates, each including three technical replicates. At the end of the treatment, plant growth parameters were recorded, and leaf samples were immediately frozen in liquid nitrogen, and stored at − 80 °C for future biochemical and molecular analyses.

### Growth parameters

Root and shoot tissues from 10 to 12 seedlings per treatment were collected to measure fresh weights (FW). Samples were then dried at 70 °C for 48 h to determine dry weight (DW). In addition, primary root length and shoot length were measured to assess growth performance.

### Cadmium content

Leaf samples were oven-dried at 70 °C for 48 h and ground into a fine powder using a mortar and pestle. Approximately 0.2 g of the material was subjected to microwave-assisted acid digestion following Radojević and Bashkin (1999). Cadmium concentrations were subsequently determined using inductively coupled plasma optical emission spectrometry (ICP-OES; Agilent 5100, USA).

### Biochemical analyses

#### Photosynthetic pigment contents

Leaf tissues (0.3 g) were homogenized in 3 ml of 80% (v/v) acetone to determine photosynthetic pigment contents. The homogenate was centrifuged, and the absorbance of the supernatant was measured at 470, 645, and 663 nm using a spectrophotometer. Total chlorophyll and carotenoid contents were calculated following Sumanta et al. (2014).

#### Hydrogen peroxide and malondialdehyde contents

Hydrogen peroxide (H_2_O_2_) and malondialdehyde (MDA) contents were determined by homogenizing 0.3 g of leaf tissue in 3 ml of ice-cold 0.1% (w/v) trichloroacetic acid (TCA). The homogenate was centrifuged at 12 000 rpm for 15 min at 4 °C, and the supernatant was used for the quantification of both H_2_O_2_ and MDA. Hydrogen peroxide content was measured spectrophotometrically at 390 nm based on triiodide formation following potassium iodide oxidation (Velikova et al. 2000). Malondialdehyde content was determined using the thiobarbituric acid (TBA) assay (Du and Bramlage 1992).

#### Antioxidant enzyme assays

Frozen leaf tissue (0.3 g) was homogenized in 3 ml of ice-cold phosphate buffer (0.05 M, pH 7.0) containing 1 mM disodium EDTA and 2% (w/v) polyvinylpyrrolidone. Homogenates were centrifuged at 11 000 rpm for 15 min at 4 °C, and the resulting supernatants were used for protein quantification and enzyme activity assays. Total soluble protein content was determined using the Bradford (1976) method. Activities of superoxide dismutase (SOD; EC 1.15.1.1) and catalase (CAT; EC 1.11.1.6) were quantified using plant specific commercial ELISA kits (SunRed, China), following the manufacturer’s instructions. Guaiacol peroxidase (GPX; EC 1.11.1.7) and ascorbate peroxidase (APX; EC 1.11.1.11) activities were assayed according to Scebba et al. (2001) and Nakano and Asada (1981), respectively.

#### Free proline level

For proline determination, leaf tissue (0.3 g) was extracted with 5 ml of 3% (w/v) sulfosalicylic acid and filtered through Whatman No. 2 paper. An aliquot (1 ml) of the filtrate was mixed with 2 ml of acid–ninhydrin reagent and incubated at 100 °C for 30 min. After cooling in an ice bath, absorbance was measured at 508 nm (Shabnam et al. 2016), and free proline content was estimated using an L-proline standard curve.

#### Total phenolic and flavonoid contents

For the determination of total phenolic and flavonoid contents, frozen leaf samples (0.3 g) were homogenized in 3 ml of absolute methanol and centrifuged at 13 000 rpm for 10 min at 4 °C. The supernatants were used for following analyses. Total phenolic content was determined according to Maksimović and Živanović (2012) and expressed as caffeic acid equivalents (CAE). Total flavonoid content was measured as described by Shubhangi et al. (2017) and expressed as µg quercetin equivalents (QE) g^− 1^ FW.

### Molecular analysis

Total RNA was isolated from leaf tissues using the GeneJET Plant RNA Purification Kit (Thermo Fisher Scientific, Germany), based on the manufacturer’s instructions. RNA concentration and purity were assessed spectrophotometrically, and RNA integrity was verified by agarose gel electrophoresis. To eliminate residual genomic DNA contamination, RNA samples were treated with DNase I (Thermo Fisher Scientific, Germany). First-strand cDNA was synthesized from 1 µg of RNA using the RevertAid First Strand cDNA Synthesis Kit (Thermo Fisher Scientific, Germany). Primer sequences, gene accession numbers, optimal annealing temperatures, and references of the selected antioxidant-related genes (*CSD5*, *MSD1*, *FSD3*, *CAT1*, *APX1*, and *POD*) in *Glycine max* are listed in Table S1. Primers were designed with MacVector Pro 18 software (USA), considering GC content and absence of primer–dimers. qRT-PCR was conducted using 2×AMPIGENE qPCR Green Mix (Enzo Life Sciences, USA) on a StepOnePlus™ Real-Time PCR System (Thermo Fisher Scientific, Germany). The *Actin6* gene was used as the internal reference gene. Amplification specificity was verified by melting curve analysis. Relative transcript abundance was calculated using the 2^⁻ΔΔCT^ method (Livak and Schmittgen 2001).

### Statistical analysis

Prior to statistical analysis, data normality and variance homogeneity were assessed using the Shapiro–Wilk and Bartlett’s tests, respectively. Differences among treatments were evaluated by one-way analysis of variance (ANOVA), followed by Duncan’s multiple range test. Statistical significance was set at *p* < 0.05.

## Results

### Growth and Cd content

**Fig. 1:**
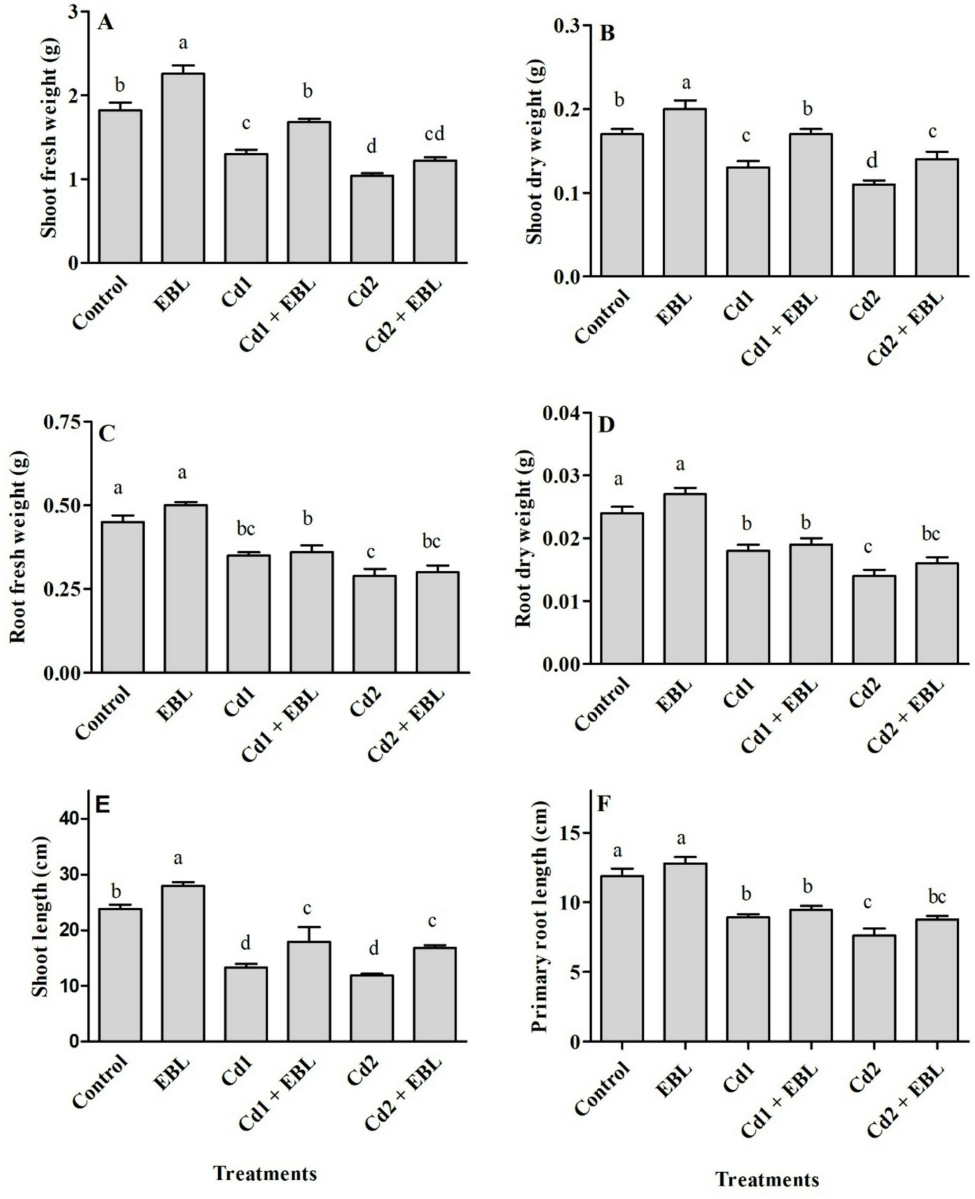
Effects of foliar-applied 24-Epibrassinolide on shoot fresh weight (**A**), shoot dry weight (**B**), root fresh weight (**C**), root dry weight (**D**), shoot length (**E**), and primary root length (**F**) of soybean seedlings under Cd stress. Cd1 and Cd2 correspond to 20 and 40 mg L^− 1^ Cd, respectively. Values are presented as means ± SEM (*n* = 10–12). Different letters above the bars indicate statistically significant differences among treatments according to Duncan’s multiple range test (*p* < 0.05)

**Fig. 2:**
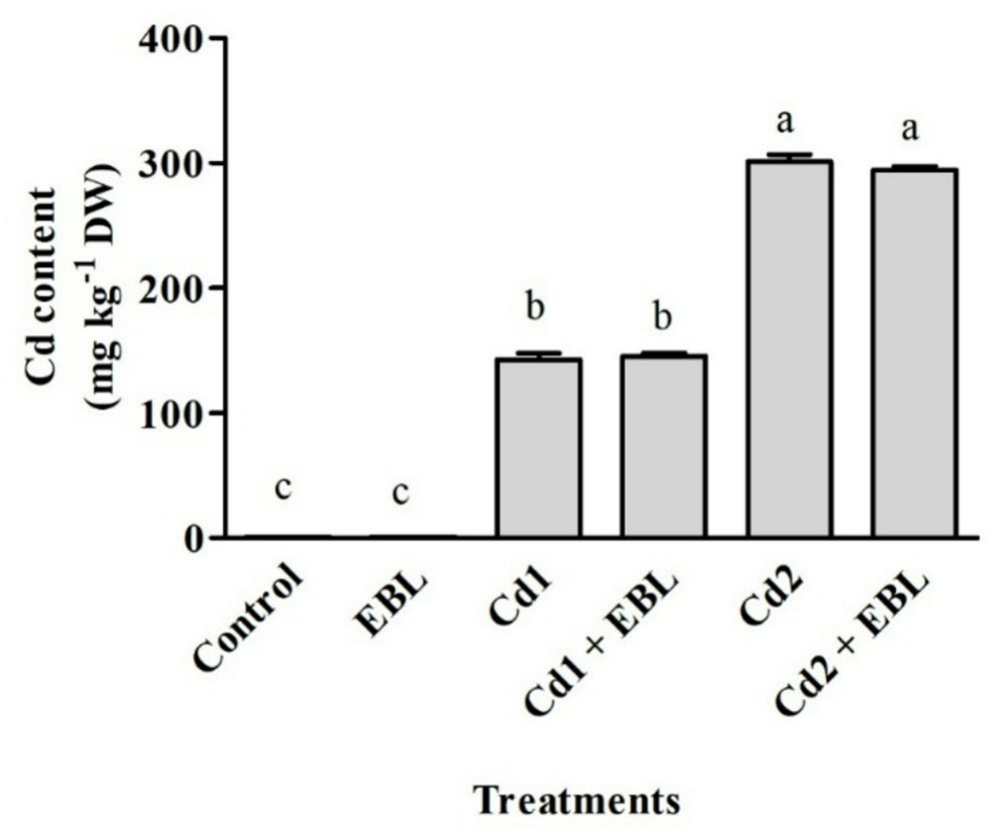
Effects of foliar-applied 24-Epibrassinolide on Cd accumulation in the leaves of soybean seedlings under Cd stress. Cd1 and Cd2 correspond to 20 and 40 mg L^− 1^ Cd, respectively. Values are presented as means ± SEM (*n* = 3). Different letters above the bars indicate statistically significant differences among treatments according to Duncan’s multiple range test (*p* < 0.05)

Cadmium-induced reductions were observed at both 20 and 40 mg L^− 1^ in shoot and root fresh and dry weights (Fig. 1A–D), as well as in shoot and primary root lengths (Fig. 1E and F). Exogenous EBL application, either alone or in combination with Cd, significantly improved shoot growth parameters (Fig. 1A, B and E). Cadmium exposure resulted in 185.3- and 390.9-fold increases of the metal in soybean seedling leaves at 20 and 40 mg L^− 1^, respectively (Fig. 2).

### Biochemical parameters

**Fig. 3:**
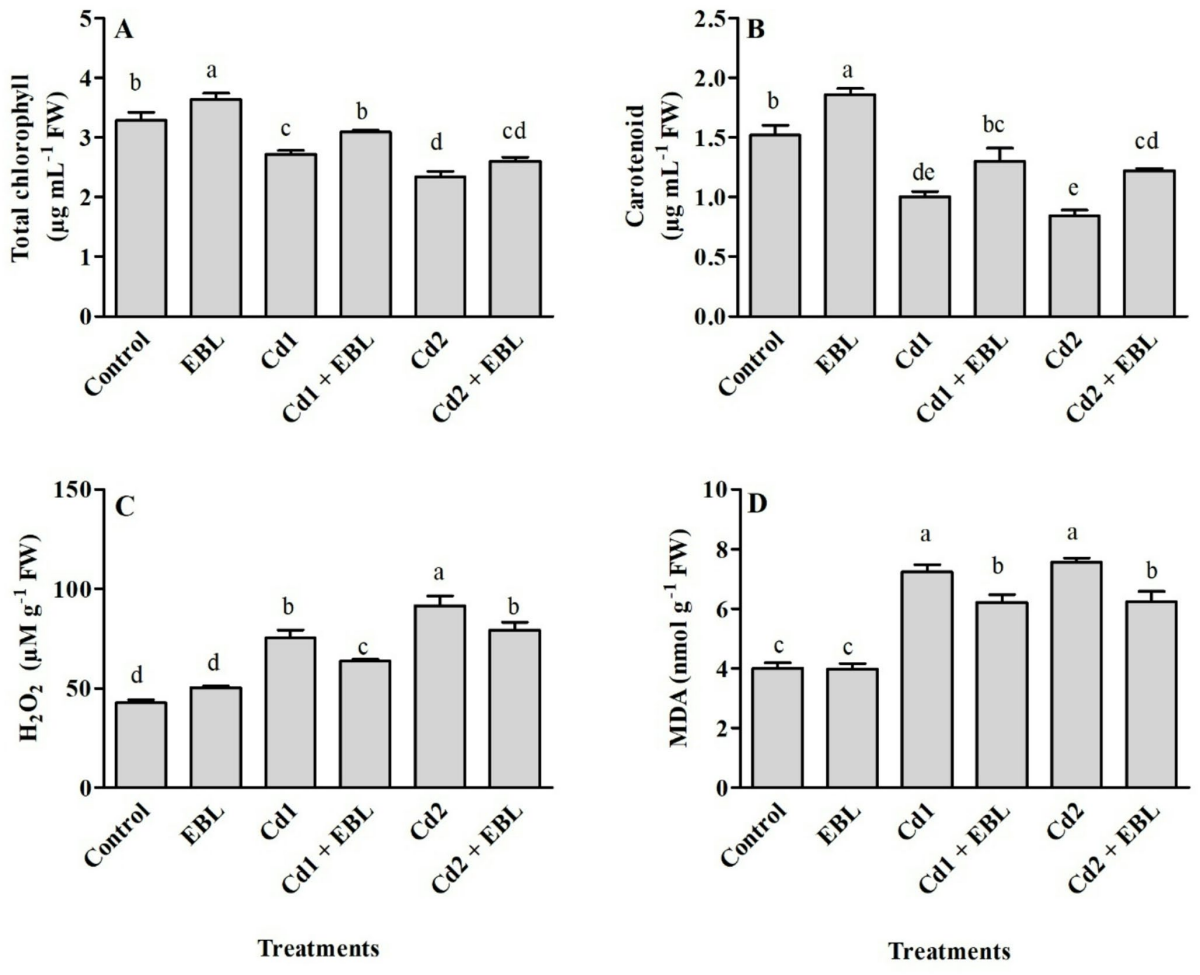
Effects of foliar-applied 24-Epibrassinolide on total chlorophyll (**A**), carotenoid (**B**), hydrogen peroxide (H_2_O_2_) (**C**), and malondialdehyde (MDA) (**D**) contents in the leaves of soybean seedlings under Cd stress. Cd1 and Cd2 correspond to 20 and 40 mg L^− 1^ Cd, respectively. Values are presented as means ± SEM (*n* = 4). Different letters above the bars indicate statistically significant differences among treatments according to Duncan’s multiple range test (*p* < 0.05)

**Fig. 4:**
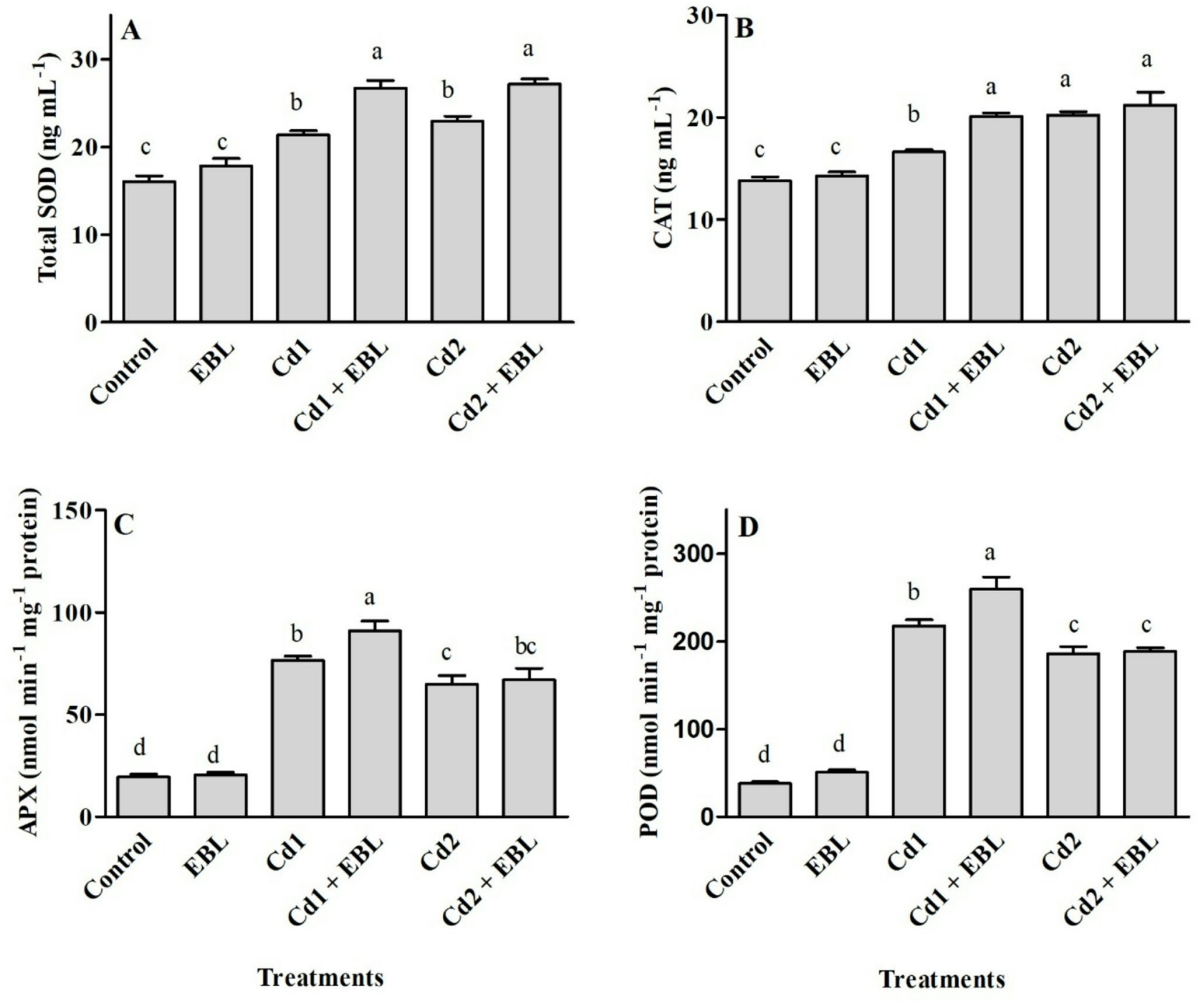
Effects of foliar-applied 24-Epibrassinolide on the activities of total superoxide dismutase (SOD) (**A**), catalase (CAT) (**B**), ascorbate peroxidase (APX) (**C**), and peroxidase (POD) (**D**) in the leaves of soybean seedlings under Cd stress. Cd1 and Cd2 correspond to 20 and 40 mg L^− 1^ Cd, respectively. Values are presented as means ± SEM (*n* = 4). Different letters above the bars indicate statistically significant differences among treatments according to Duncan’s multiple range test (*p* < 0.05)

**Fig. 5:**
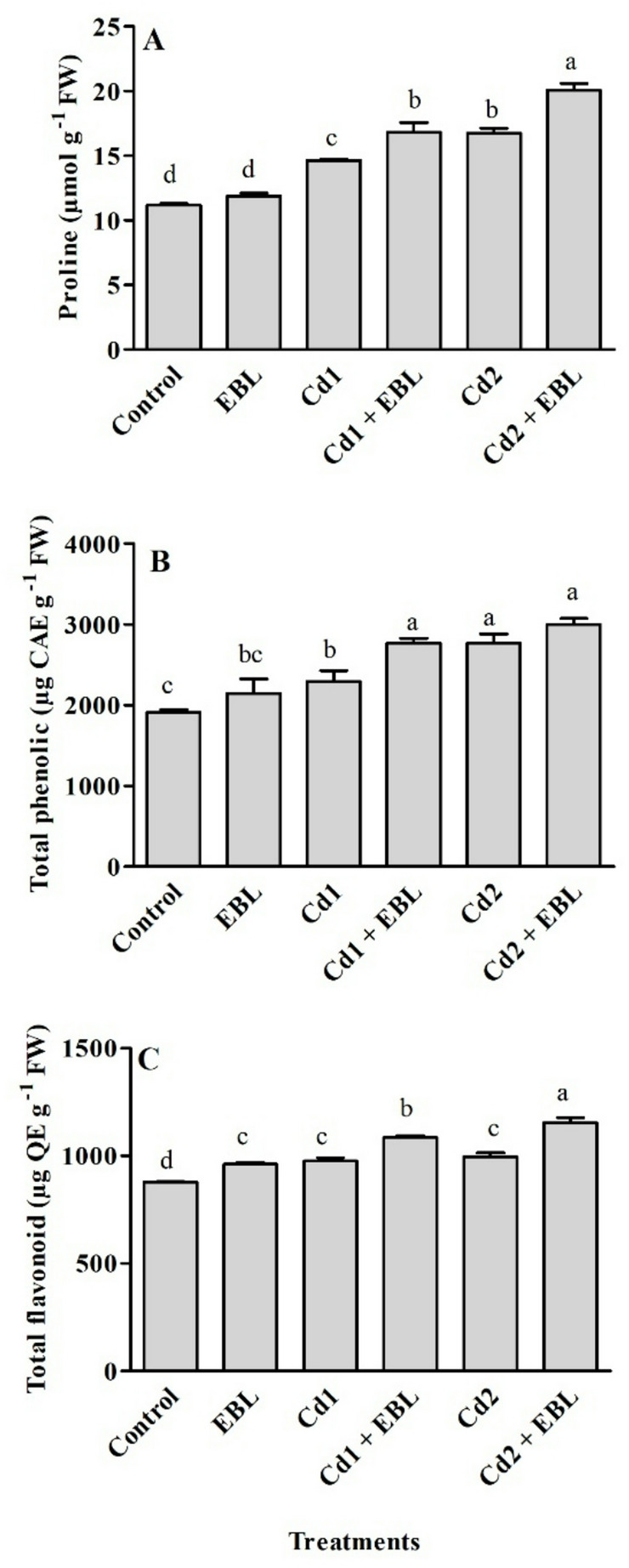
Effects of foliar-applied 24-Epibrassinolide on proline (**A**), total phenolic (**B**), and total flavonoid (**C**) contents in the leaves of soybean seedlings under Cd stress. Cd1 and Cd2 correspond to 20 and 40 mg L^− 1^ Cd, respectively. Values are presented as means ± SEM (*n* = 4). Different letters above the bars indicate statistically significant differences among treatments according to Duncan’s multiple range test (*p* < 0.05)

Total chlorophyll and carotenoid contents in leaves responded similarly to Cd and/or EBL treatments (Fig. 3A and B). Cadmium stress decreased chlorophyll and carotenoid levels, while EBL treatment alone enhanced these pigments compared with the control. Co-application of EBL with 20 mg L^− 1^ Cd significantly mitigated chlorophyll loss, restoring levels to those comparable to the control (Fig. 3A). Furthermore, carotenoid content was enhanced by EBL under both Cd concentrations (Fig. 3B). A marked increase in H_2_O_2_ and MDA contents were observed under Cd treatments compared with the untreated samples. Foliar application of EBL under Cd stress reduced these oxidative stress markers relative to Cd treatment alone (Fig. 3C and D). SOD activity was similarly enhanced at 20 and 40 mg L^− 1^ Cd, whereas CAT activity increased progressively with rising Cd concentration, showing a dose-dependent response (Fig. 4A and B). Both Cd treatments enhanced APX and GPX activities, with 20 mg L^− 1^ inducing the highest increase among the tested concentrations (Fig. 4C and D). Exogenous EBL further enhanced SOD activity under Cd treatments, whereas stimulatory effects on CAT, APX, and GPX were observed only at 20 mg L^− 1^ Cd (Fig. 4). Free proline, total flavonoid, and phenolic contents significantly increased in response to 20 and 40 mg L^− 1^ Cd treatments compared with the control (Fig. 5). Co-application of EBL with Cd further elevated proline and flavonoid levels beyond those observed in the control and Cd-only treatments (Fig. 5A and C). The 20 mg L^− 1^ Cd + EBL treatment also increased total phenolic content compared with 20 mg L^− 1^ Cd alone (Fig. 5B).

### Gene expression

**Fig. 6:**
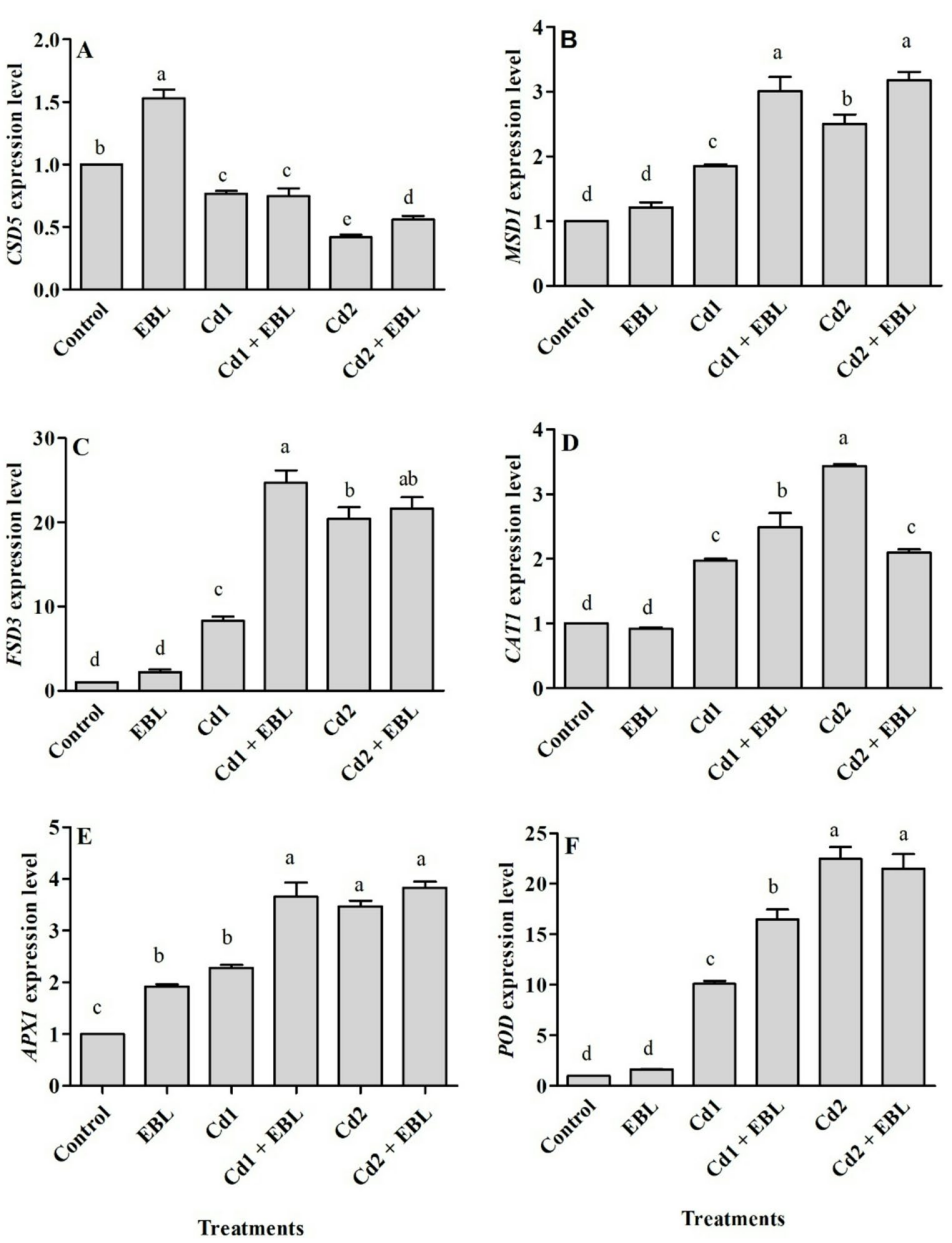
Effects of foliar-applied 24-Epibrassinolide on the relative expression levels of *CSD5* (**A**), *MSD1* (**B**), *FSD3* (**C**), *CAT1* (**D**), *APX1* (**E**), and *POD* (**F**) genes in the leaves of soybean seedlings under Cd stress. Cd1 and Cd2 correspond to 20 and 40 mg L^− 1^ Cd, respectively. Values are presented as means ± SEM (*n* = 3). Different letters above the bars indicate statistically significant differences among treatments according to Duncan’s multiple range test (*p* < 0.05)

Under Cd stress, foliar EBL application significantly modulated the transcription of antioxidant-related genes in soybean leaves (Fig. 6). The examined *SOD* isoforms (*CSD5*, *MSD1*, and *FSD3*) exhibited distinct expression profiles in response to Cd and/or EBL treatment. *CSD5* expression increased following EBL application alone, whereas both 20 and 40 mg L^− 1^ Cd treatments suppressed its expression in leaves. Notably, under 40 mg L^− 1^ Cd, EBL markedly elevated *CSD5* transcript levels relative to the corresponding Cd-only treatment (Fig. 6A). *MSD1* expression increased in a dose-dependent manner (1.8- and 2.5-fold at 20 mg L^− 1^ and 40 mg L^− 1^, respectively), and this induction was further enhanced by EBL at both concentrations (Fig. 6B). Among the analyzed genes, *FSD3* showed the most pronounced transcriptional response. Specifically, 20 and 40 mg L^− 1^ Cd induced 8.3- and 20.4-fold increases, respectively, in *FSD3* mRNA levels. Co-application of 20 mg L^− 1^ Cd with EBL led to a 24.7-fold increase in *FSD3* transcript abundance in leaves (Fig. 6C).

Following 10 days of Cd exposure, *CAT1*, *APX1*, and *POD* expression showed significant increases in response to rising Cd concentrations (Fig. 6D–F). EBL further enhanced *CAT1* transcript levels by 26.3% at 20 mg L^− 1^ Cd, whereas it slightly reduced *CAT1* expression at 40 mg L^− 1^ Cd compared with the corresponding Cd-alone treatment (Fig. 6D). *APX1* transcript levels were elevated 1.9-fold in response to EBL alone (Fig. 6E). *APX1* and *POD* expression in leaves of seedlings exposed to 20 mg L^− 1^ Cd were significantly stimulated by EBL (Fig. 6E and F).

## Discussion

Cadmium exposure negatively affected soybean seedling development, leading to reduced shoot and root growth. The inhibitory effects of Cd on root development may be attributed to enhanced monolignol biosynthesis, which promotes lignin deposition, reinforces root cell walls, and consequently restricts cell elongation and root growth (Finger-Teixeira et al. 2010). In addition, elevated Cd concentrations also disrupt nutrient uptake and water acquisition, further limiting overall plant growth and development (Gomes et al. 2013). In contrast, the observed improvement in shoot growth following foliar EBL application under Cd stress reveals a potential role of EBL in alleviating Cd-induced growth inhibition. This growth-promoting effect is consistent with previous reports (Anuradha and Rao 2009; Alam et al. 2021). Exogenous EBL has been shown to enhance photosynthetic performance in metal stressed plants, which may substantially contribute to increased biomass production under Cd stress (Ali et al. 2008). Moreover, BRs stimulate the biosynthesis of organic acids and help maintain the functionality of vascular tissues responsible for efficient water and nutrient transport from roots to the apical meristem (Vardhini et al. 2010), thereby supporting sustained growth even under adverse conditions.

In the present study, Cd accumulation in soybean leaves increased with rising Cd concentrations, whereas foliar application of EBL had no significant effect on Cd levels. Earlier studies have reported a reduction in Cd accumulation following EBL treatment (Santos et al. 2018; Huang et al. 2024). This discrepancy may reflect differences in EBL application method, timing, and concentration, as well as plant species, genotype, or exposure duration. Under the conditions of this study, these results suggest that EBL’s protective effect in soybean is mainly mediated by enhanced antioxidant defences.

Chlorophyll is essential for photosynthesis, and its concentration in leaves represents a key determinant of overall plant growth (Tantray et al. 2020). Similarly, carotenoids act as potent antioxidants in plant cells by mitigating reactive oxygen species (ROS) accumulation and protecting biomolecules from oxidative stress induced damage (Sharma et al. 2012). Consistent with the present findings, several studies have reported that Cd exposure reduces pigment levels in soybean (Dražić et al. 2004; Xue et al. 2013; Pérez Chaca et al. 2014). Liu et al. (2023) proposed that this reduction may result from competitive interactions of Cd with magnesium, iron, and zinc at the binding sites of key photosynthetic enzymes, including fructose-1,6-bisphosphate aldolase, RuBisCo, and carbonic anhydrase. Comparable increases in pigment levels following EBL application under Cd stress have been reported in Cd-stressed *Lycopersicum esculentum* (Hasan et al. 2011). Additionally, foliar application of 1 µM EBL increased chlorophyll content in *Vigna angularis* leaves under Cd stress, an effect associated with the upregulation of chlorophyll biosynthesis–related genes and the maintenance of photosynthetic efficiency (Chen et al. 2025).

Soybean leaves exhibited a concentration-dependent increase in H_2_O_2_ accumulation and lipid peroxidation (MDA) under Cd treatment. Chmielowska-Bąk et al. (2017) demonstrated that Cd exposure in soybean seedlings activates NADPH oxidase, leading to elevated ROS production. Foliar application of EBL reduced both H_2_O_2_ and MDA levels, indicating that EBL enhances antioxidant capacity and helps maintain membrane stability under Cd-induced oxidative stress. Consistent with these observations, Shah et al. (2019) reported that EBL alleviated Cd toxicity in *Cucumis sativus* by modulating alternative oxidase (AOX), a key component of mitochondrial ROS detoxification, while simultaneously increasing ethylene production and reducing H_2_O_2_ accumulation. Similarly, brassinolide treatment significantly decreased oxidative stress markers in Cd-exposed *Wolffia arrhiza* seedlings (Chmur and Bajguz 2025). Cadmium-induced ROS accumulation is known to activate the antioxidant system responsible for cellular detoxification. The observed increases in SOD, CAT, APX, and POD activities, along with elevated H_2_O_2_ levels, reflected a coordinated activation of the antioxidative network in response to Cd stress, consistent with previous reports of enhanced antioxidant enzyme activities under similar Cd exposure (Gong et al. 2023). In line with our findings, earlier studies have shown that BRs stimulate key antioxidant enzymes, reducing Cd-induced oxidative damage in various plant species, including *Brassica juncea* (Hayat et al. 2007) and *Lycopersicon esculentum* (Hasan et al. 2011). This EBL-mediated tolerance to heavy metal stress is linked to upregulation of antioxidant system–related genes, providing a mechanistic explanation for the observed increase in enzymatic activities under Cd stress (Bajguz 2000; Sharma et al. 2016; Jia et al. 2021).

Plants accumulate osmoprotective metabolites, particularly proline, which contributes to osmotic adjustment by increasing cytosolic solutes and lowering cellular osmotic potential under stress (Posmyk et al. 2009). Wang et al. (2025) demonstrated that Cd-induced upregulation of *GmP5CS* in vegetable soybean enhances proline accumulation, thereby supporting Cd tolerance. Treatment with Cd in the present study increased proline levels in soybean leaves, which were further elevated by foliar EBL. Similar trends have been reported in *Cucumis sativus* (Shah et al. 2020) and *Brassica juncea* (Alam et al. 2020) under Cd stress. Moreover, Surgun-Acar and Zemheri-Navruz (2022) reported that EBL enhanced proline accumulation and upregulated *P5CS1* and *P5CS2*, the genes encoding the proline biosynthetic enzyme P5CS, in *Arabidopsis thaliana* exposed to manganese stress.

Phenolic compounds play a crucial role in plant defence against heavy metal toxicity, including Cd, by mitigating oxidative damage within plant cells (Goncharuk and Zagoskina 2023). Flavonoids, a diverse class of plant phenolics, chelate metal ions, thereby reducing hydroxyl radical formation and maintaining cellular stability (Fernandez et al. 2002). The present results align with those of Tadjouri et al. (2022), who reported that exposure of soybean seedlings to varying Cd concentrations led to significant increases in total phenolic and flavonoid contents in both root and leaf tissues. In this study, exogenous EBL enhanced the accumulation of phenolic and flavonoid compounds in soybean leaves under Cd stress, pointing to strengthened non-enzymatic antioxidant defences. Similarly, in *Citrullus lanatus*, EBL upregulated key genes in the phenylpropanoid pathway (*PAL*, *4CL*, *CCR*, and *CCoAOMT*), increased related enzyme activities, and elevated phenolic, flavonoid, and lignin levels ( Liu et al. 2023)

To further elucidate the regulatory effects of foliar-applied EBL on the antioxidant system, the expression of genes encoding key antioxidant enzymes (*CSD5*, *MSD1*, *FSD3*, *CAT1*, *APX1*, and *POD*) was analyzed. Cadmium exposure induced isoform-specific modulation of *SOD* gene expression, with *MSD1* and *FSD3* were upregulated, whereas *CSD5* was downregulated. These contrasting patterns reflect the association of SOD regulation with their distinct subcellular localization, as Cu/Zn-SOD (*CSD5*), Fe-SOD (*FSD3*), and Mn-SOD (*MSD1*) are predominantly localized in the cytosol, chloroplasts, and mitochondria, respectively (Mittler 2002; Gill and Tuteja 2010). This compartment-specific distribution may support complementary regulation of superoxide detoxification under stress conditions. Moreover, *CAT1*, *APX1*, and *POD* transcripts increased at both Cd concentrations, indicating coordinated transcriptional activation of antioxidant defences. In soybean seedlings, El-Esawi et al. (2020) reported a positive correlation between antioxidant enzyme activities and the expression of their corresponding genes (*SOD*, *CAT*, *APX*, and *POD*), all upregulated under Cd stress. The same study also observed enhanced transcripts of genes involved in flavonoid and proline biosynthesis. Foliar application of EBL further upregulated the expression of most antioxidant-related genes under Cd stress, except for *CSD5*, particularly at the lower Cd concentration. Similar BR-induced increases in antioxidant gene expression have been reported in *Arabidopsis thaliana* under arsenic stress (Surgun-Acar and Zemheri-Navruz 2019) and in *Triticum aestivum* under both mercury (Işkil et al. 2022) and Cd stress (Shah et al. 2023). In response to Cd exposure, H_2_O_2_ acts as a signaling molecule, activating mitogen-activated protein kinase (MAPK)-dependent pathways and promoting the transcription of antioxidant genes such as *SOD*, *CAT*, and *APX*, thereby contributing to stress adaptation (Lin and Aarts 2012; Jagodzik et al. 2018). Notably, BRs have also been shown to activate the MAPK cascade, enhancing BR biosynthesis and stimulating the production of other growth-related phytohormones (Planas-Riverola et al. 2019).

## Conclusion

Cadmium exposure induced oxidative stress in soybean seedlings, as evidenced by growth inhibition, reduced pigment content, and elevated oxidative stress markers, reflecting disrupted cellular homeostasis. Foliar application of EBL mitigated these adverse effects by enhancing antioxidant defences at both physiological and biochemical levels, while transcriptional modulation of antioxidant-related genes suggests a coordinated regulatory response. Collectively, these findings highlight the potential of foliar EBL to enhance antioxidant capacity and improve soybean tolerance to Cd stress, although further field studies are needed to confirm its practical efficacy.

## Supplementary Information

Below is the link to the electronic supplementary material.

Supplementary Material 1

## Data Availability

All data generated or analyzed during this study are included in this published article.

## References

[CR1] Alam P, Kaur Kohli S, Al Balawi T, Altalayan FH, Alam P, Ashraf M, Bhardwaj R, Ahmad P (2020) Foliar application of 24-Epibrassinolide improves growth, ascorbate-glutathione cycle, and glyoxalase system in brown mustard (*Brassica juncea* (L.) Czern.) under cadmium toxicity. Plants 9(11):1487. 10.3390/plants9111487 33158232 10.3390/plants9111487 PMC7694298

[CR2] Alam P, Balawi TA, Ashraf M, Ahmad P (2021) 24-Epibrassinolide (EBR) reduces oxidative stress damage induced by cadmium toxicity by restricting cd uptake and modulating some key antioxidant enzymes in maize plants. Pak J Bot 53(1):59–66. 10.30848/PJB2021-1(27)

[CR3] Ali B, Hayat S, Fariduddin Q, Ahmad A (2008) 24-Epibrassinolide protects against the stress generated by salinity and nickel in *Brassica juncea*. Chemosphere 72(9):1387–1392. 10.1016/j.chemosphere.2008.04.012 18499221 10.1016/j.chemosphere.2008.04.012

[CR4] Anuradha S, Rao SSR (2009) Effect of 24-epibrassinolide on the photosynthetic activity of radish plants under cadmium stress. Photosynthetica 47:317–320. 10.1007/s11099-009-0050-3

[CR5] Aserse AA, Räsänen LA, Aseffa F, Hailemariam A, Lindström K (2012) Phylogenetically diverse groups of Bradyrhizobium isolated from nodules of *Crotalaria spp*., *Indigofera spp*., *Erythrina brucei* and *Glycine max* growing in Ethiopia. Mol Phylogenet Evol 65(2): 595–609. 10.1016/j.ympev.2012.07.008 10.1016/j.ympev.2012.07.008 22842091

[CR6] Bajguz A (2000) Effect of brassinosteroids on nucleic acids and protein content in cultured cells of *Chlorella vulgaris*. Plant Physiol Biochem 38(3):209–215. 10.1016/S0981-9428(00)00733-6

[CR7] Balestrasse KB, Gardey L, Gallego SM, Tomaro ML (2001) Response of antioxidant defence system in soybean nodules and roots subjected to cadmium stress. Funct Plant Biol 28(6):497–504. 10.1071/PP00158

[CR8] Bradford MM (1976) A rapid and sensitive method for the quantitation of microgram quantities of protein utilizing the principle of protein-dye binding. Anal Biochem 72(1–2):248–254 942051 10.1016/0003-2697(76)90527-3

[CR9] Chakraborty N, Ganguly R, Sarkar A, Dasgupta D, Sarkar J, Acharya K, Burachevskaya M, Minkina T, Keswani C (2025) Multifunctiomal Role of Brassinosteroids in Plant Growth, Development, and Defense. J Plant Growth Reg 44:2627–2640. 10.1007/s00344-024-11593-4

[CR10] Chen S, Tang Z, Hou J, Gao J, Li X, Zhang Y, Zhao Q (2025) 2,4-Epibrassinolide Mitigates Cd Stress by Enhancing Chloroplast Structural Remodeling and Chlorophyll Metabolism in *Vigna angularis* Leaves. Biology 14(6):674. 10.3390/biology14060674 40563925 10.3390/biology14060674 PMC12189853

[CR11] Chmielowska-Bąk J, Lefèvre I, Lutts S, Deckert J (2013) Short term signaling responses in roots of young soybean seedlings exposed to cadmium stress. J Plant Physiol 170(18):1585–1594. 10.1016/j.jplph.2013.06.019 23942356 10.1016/j.jplph.2013.06.019

[CR12] Chmielowska-Bąk J, Arasimowicz-Jelonek M, Izbianska K, Frontasyeva M, Zinicovscaia I, Guiance-Varela C, Deckert J (2017) NADPH oxidase is involved in regulation of gene expression and ROS overproduction in soybean (*Glycine max* L.) seedlings exposed to cadmium. Acta Soc Bot Pol 86(2):3551. 10.5586/asbp.3551

[CR13] Chmur M, Bajguz A (2025) Comparative Efficacy of Melatonin and Brassinolide in Mitigating the Adverse Effects of Cadmium on *Wolffia arrhiza*. Int J Mol Sci 26(2):692. 10.3390/ijms26020692 39859406 10.3390/ijms26020692 PMC11765764

[CR14] Dražić G, Mihailović N, Stojanović Z (2004) Cadmium toxicity: the effect on macro-and micro-nutrient contents in soybean seedlings. Biol Plant 48(4):605–607. 10.1023/B:BIOP.0000047160.79306.b7

[CR15] Du Z, Bramlage WJ (1992) Modified thiobarbituric acid assay for measuring lipid oxidation in sugar-rich plant tissue extracts. J Agric Food Chem 40(9):1566–1570. 10.1021/jf00021a018

[CR16] El-Esawi MA, Elkelish A, Soliman M, Elansary HO, Zaid A, Wani SH (2020) *Serratia marcescens* BM1 enhances cadmium stress tolerance and phytoremediation potential of soybean through modulation of osmolytes, leaf gas exchange, antioxidant machinery, and stress-responsive genes expression. Antioxidants 9(1):43. 10.3390/antiox9010043 31947957 10.3390/antiox9010043 PMC7023057

[CR17] Fernandez MT, Mira ML, Florêncio MH, Jennings KR (2002) Iron and copper chelation by flavonoids: an electrospray mass spectrometry study. J Inorg Biochem 92(2):105–111. 10.1016/S0162-0134(02)00511-1 12459155 10.1016/s0162-0134(02)00511-1

[CR18] Ferreira RR, Fornazier RF, Vitória AP, Lea PJ, Azevedo RA (2006) Changes In Antioxidant Enzyme Activities In Soybean Under Cadmium Stress. J Plant Nutr 25(2):327–342. 10.1081/PLN-100108839

[CR19] Finger-Teixeira A, Ferrarese M, de LL, Soares AR, da Silva D, Ferrarese-Filho Osvaldo O (2010) Cadmium-induced lignification restricts soybean root growth. Ecotoxicol Environ Saf 73(8):1959–1964. 10.1016/j.ecoenv.2010.08.021 20817298 10.1016/j.ecoenv.2010.08.021

[CR20] Gill SS, Tuteja N (2010) Reactive oxygen species and antioxidant machinery in abiotic stress tolerance in crop plants. Plant Physiol Biochem 48(12):909–930. 10.1016/j.plaphy.2010.08.016 20870416 10.1016/j.plaphy.2010.08.016

[CR21] Gomes MP, Marques TCLLSM, Soares AM (2013) Cadmium effects on mineral nutrition of the Cd hyperaccumulator *Pfaffia glomerata*. Biologia 68(2):223–230. 10.2478/s11756-013-0005-9

[CR22] Goncharuk EA, Zagoskina NV (2023) Heavy metals, their phytotoxicity, and the role of phenolic antioxidants in plant stress responses with focus on cadmium. Molecules 28(9):3921. 10.3390/molecules28093921 37175331 10.3390/molecules28093921 PMC10180413

[CR23] Gong Z, Duan Y, Liu D, Zong Y, Zhang D, Shi X, Hao X, Li P (2023) Physiological and transcriptome analysis of response of soybean (*Glycine max*) to cadmium stress under elevated CO_2_ concentration. J Hazard Mater 448:130950. 10.1016/j.jhazmat.2023.130950 36860078 10.1016/j.jhazmat.2023.130950

[CR24] Hasan SA, Hayat S, Ahmad A (2011) Brassinosteroids protect photosynthetic machinery against the cadmium induced oxidative stress in two tomato cultivars. Chemosphere 84(10):1446–1451. 10.1016/j.chemosphere.2011.04.047 21565386 10.1016/j.chemosphere.2011.04.047

[CR25] Hayat S, Ali B, Hasan SA, Ahmad A (2007) Brassinosteroid enhanced the level of antioxidants under cadmium stress in *Brassica juncea*. Environ Exp Bot 60(1):33–41. 10.1016/j.envexpbot.2006.06.002

[CR26] Hoagland DR, Arnon DI (1938) The water culture method for growing plants without soil. Calif Agric Exper Stat Circ 3:346–347

[CR27] Huang Q, Zhang Y, Liu S, Wang H, Gao F, Shao G (2024) 24-epibrassinolide mitigates cadmium toxicity in rice plants by activating the plant detoxification system and regulating genes expression involved in Cd/Fe uptake and translocation. Plant Stress 12:100485. 10.1016/j.stress.2024.100485

[CR28] Ikhajiagbe B, Ogwu MC, Lato NF (2021) Growth and yield responses of soybean (*Glycine max* [L.] Merr.) accessions after exposure to cadmium. Vegetos 34:107–118. 10.1007/s42535-021-00189-y

[CR29] Ingkar A, Gulden K, Zargar M, Gang HY, Dana S, Bekzak A, Aliya B, Gani S (2025) Comparative Assessment of Different Soybean Genotypes (*Glycine max* [L.] Merr.) for Early Maturity and Cold Resistance. ES Food Agrofor 21:1731. 10.30919/faf1731

[CR30] Işkil R, Surgun-Acar Y, Çatav ŞS, Zemheri-Navruz F, Erden Y (2022) Mercury toxicity affects oxidative metabolism and induces stress responsive mechanisms in wheat (*Triticum aestivum* L). Physiol Mol Biol Plants 28(4):911–920. 10.1007/s12298-022-01171-x 35592475 10.1007/s12298-022-01171-x PMC9110583

[CR31] Jagodzik P, Tajdel-Zielińska M, Cieśla A, Marczak M, Ludwikow A (2018) Mitogen activated protein kinase cascades in plant hormone signaling. Front Plant Sci 9:1387. 10.3389/fpls.2018.01387 30349547 10.3389/fpls.2018.01387 PMC6187979

[CR32] Jia C, Zhao S, Bao T, Zhao P, Peng K, Guo Q, Gao X, Qin J (2021) Tomato BZR/BES transcription factor SIBZR1 positively regulates BR signaling and salt stress tolerance in tomato and *Arabidopsis*. Plant Sci 302:110719. 33288025 10.1016/j.plantsci.2020.110719

[CR33] Kumari S, Dambale AS, Samantara R, Jincy M, Bains G (2025) Introduction, History, Geographical Distribution, Importance, and Uses of Soybean (*Glycine max* L). In: Singh KP, Singh NK, Aravind T (eds) Soybean Production Technology. Springer, Singapore. 10.1007/978-981-97-8677-0_1

[CR34] Li S, Zheng H, Lin L, Wang F, Sui N (2021) Roles of brassinosteroids in plant growth and abiotic stress response. Plant Growth Regul 93:29–38. 10.1007/s10725-020-00672-7

[CR35] Lin YF, Aarts MG (2012) The molecular mechanism of zinc and cadmium stress response in plants. Cell Mol Life Sci 69(19):3187–3206. 10.1007/s00018-012-1089-z 22903262 10.1007/s00018-012-1089-z PMC11114967

[CR36] Liu J, Ni J, Mo A, Fan X, Jiang Y, Xie H, Hu J, Zhu Y, Peng C, Yang F (2023) Cadmium affects the growth, antioxidant capacity, chlorophyll content, and homeostasis of essential elements in soybean plants. S Afr J Bot 162:604–610. 10.1016/j.sajb.2023.09.059

[CR37] Liu X, Zhu Q, Liu W, Zhang J (2023) Exogenous Brassinosteroid Enhances Zinc tolerance by activating the Phenylpropanoid Biosynthesis pathway in *Citrullus lanatus* L. Plant Signal Behav 18(1):2186640. 10.1080/15592324.2023.2186640 37083111 10.1080/15592324.2023.2186640 PMC10124981

[CR38] Livak KJ, Schmittgen TD (2001) Analysis of Relative Gene Expression Data Using Real-Time Quantitative PCR and the 2^ – ∆∆CT^ Method. Methods 25(4):402–408. 10.1006/meth.2001.1262 11846609 10.1006/meth.2001.1262

[CR39] Maia CF, da Silva BRS, Batista BL, Bajguz A, Lobato AKDS (2022) 24-Epibrassinolide simultaneously stimulates photosynthetic machinery and biomass accumulation in tomato plants under lead stress: Essential contributions connected to the antioxidant system and anatomical structures. Agronomy 12(9):1985. 10.3390/agronomy12091985

[CR40] Maksimović JJD, Živanović BD (2012) Quantification of the Antioxidant Activity in Salt-Stressed Tissues. In: Shabala S, Cuin T (eds) Plant Salt Tolerance, Methods in Molecular Biology, vol 913. Humana, Totowa, NJ. 10.1007/978-1-61779-986-0_16 10.1007/978-1-61779-986-0_16 22895764

[CR41] Mittler R (2002) Oxidative stress, antioxidants and stress tolerance. Trends Plant Sci 7(9):405–410. 10.1016/S1360-1385(02)02312-9 12234732 10.1016/s1360-1385(02)02312-9

[CR42] Mumtaz MA, Hao Y, Mehmood S, Shu H, Zhou Y, Jin W, Chen C, Li L, Altaf MA, Wang Z (2022) Physiological and transcriptomic analysis provide molecular insight into 24-epibrassinolide mediated Cr (VI)-toxicity tolerance in pepper plants. Environ Pollut 306:119375. 10.1016/j.envpol.2022.119375 35500717 10.1016/j.envpol.2022.119375

[CR43] Nakano Y, Asada K (1981) Hydrogen peroxide is scavenged by ascorbate-specific peroxidase in spinach chloroplasts. Plant Cell Physiol 22(5):867–880. 10.1093/oxfordjournals.pcp.a076232

[CR44] Pérez Chaca MV, Vigliocco A, Reinoso H, Molina A, Abdala G, Zirulnik F, Pedranzani H (2014) Effects of cadmium stress on growth, anatomy and hormone contents in *Glycine max* (L.) Merr. Acta Physiol Plant 36(10):2815–2826. 10.1007/s11738-014-1656-z

[CR45] Planas-Riverola A, Gupta A, Betegón-Putze I, Bosch N, Ibañes M, Caño-Delgado AI (2019) Brassinosteroid signaling in plant development and adaptation to stress. Development 146(5):dev151894. 10.1242/dev.151894 30872266 10.1242/dev.151894 PMC6432667

[CR46] Posmyk MM, Kontek R, Janas KM (2009) Antioxidant enzymes activity and phenolic compounds content in red cabbage seedlings exposed to copper stress. Ecotoxicol Environ Saf 72(2):596–602. 10.1016/j.ecoenv.2008.04.024 18801573 10.1016/j.ecoenv.2008.04.024

[CR47] Qin S, Liu H, Nie Z, Rengel Z, Gao W, Li C, Zhao P (2020) Toxicity of cadmium and its competition with mineral nutrients for uptake by plants: A review. Pedosphere 30(2):168–180. 10.1016/S1002-0160(20)60002-9

[CR48] Radojević M, Bashkin VN (1999) Practical environmental analysis. The Royal Society of Chemistry, Cornwall. 10.1039/9781847551740

[CR49] Riaz M, Kamran M, Rizwan M, Ali S, Parveen A, Malik Z, Wang X (2021) Cadmium uptake and translocation: selenium and silicon roles in Cd detoxification for the production of low Cd crops: a critical review. Chemosphere 273:129690. 10.1016/j.chemosphere.2021.129690 33524757 10.1016/j.chemosphere.2021.129690

[CR50] Rizwan M, Ali S, Zia Ur Rehman M, Rinklebe J, Tsang DCW, Bashir A, Maqbool A, Tack FMG, Ok YS (2018) Cadmium phytoremediation potential of brassica crop species: A review. Sci Total Environ 631–632:1175–1191. 10.1016/j.scitotenv.2018.03.104 29727943 10.1016/j.scitotenv.2018.03.104

[CR51] Santos LR, Batista BL, Lobato AKS (2018) Brassinosteroids mitigate cadmium toxicity in cowpea plants. Photosynthetica 56:591–605. 10.1007/s11099-017-0700-9

[CR52] Scebba F, Sebastiani L, Vitagliano C (2001) Activities of antioxidant enzymes during senescence of *Prunus armeniaca* leaves. Biol Plant 44:41–46. 10.1023/A:1017962102950

[CR53] Shabab Z, Sarada DL (2024) 24-Epibrassinolide mitigates arsenate stress in seedlings of *Oryza sativa* (IR-20) via the induction of phenylpropanoid pathway. Plant Physiol Biochem 215:109023. 10.1016/j.plaphy.2024.109023 39146914 10.1016/j.plaphy.2024.109023

[CR54] Shabnam N, Tripathi I, Sharmila P, Pardha-Saradhi P (2016) A rapid, ideal, and eco-friendlier protocol for quantifying proline. Protoplasma 253:1577–1582. 10.1007/s00709-015-0910-6 26573534 10.1007/s00709-015-0910-6

[CR55] Shah AA, Ahmed S, Yasin NA (2019) 24-epibrassinolide triggers cadmium stress mitigation in *Cucumis sativus* through intonation of antioxidant system. S Afr J Bot 127:349–360. 10.1016/j.sajb.2019.11.003

[CR56] Shah AA, Ahmed S, Abbas M, Yasin NA (2020) Seed priming with 3-epibrassinolide alleviates cadmium stress in *Cucumis sativus* through modulation of antioxidative system and gene expression. Sci Hortic 265:109203. 10.1016/j.scienta.2020.109203

[CR57] Shah T, Khan Z, Asad M, Imran A, Niazi MBK, Alsahli AA (2023) Alleviation of cadmium toxicity in wheat by strigolactone: Regulating cadmium uptake, nitric oxide signaling, and genes encoding antioxidant defense system. Plant Physiol Biochem 202:107916. 10.1016/j.plaphy.2023.107916 37595403 10.1016/j.plaphy.2023.107916

[CR58] Shahid M, Dumat C, Khalid S, Niazi NK, Antunes PMC (2017) Cadmium bioavailability, uptake, toxicity and detoxification in soil-plant system. Rev Environ Contam Toxicol 241:73–137. 10.1007/398_2016_8 27300014 10.1007/398_2016_8

[CR59] Shanmugaraj BM, Malla A, Ramalingam S (2019) Cadmium stress and toxicity in plants: An overview, Chap. 1 - Cadmium Toxicity and Tolerance in Plants, in: Hasanuzzaman M, Prasad MNV, Fujita M (Eds). Cadmium Toxicity and Tolerance in Plants, Academic Press. 1–17. 10.1016/B978-0-12-814864-8.00001-2

[CR61] Sharma I, Pati PK, Bhardwaj R (2011) Effect of 24-epibrassinolide on oxidative stress markers induced by nickel-ion in *Raphanus sativus* L. Acta Physiol Plant 33(5):1723–1735. 10.1007/s11738-010-0709-1

[CR62] Sharma P, Jha AB, Dubey RS, Pessarakli M (2012) Reactive oxygen species, oxidative damage, and antioxidative defense mechanism in plants under stressful conditions. J Bot 2012: 217037. 10.1155/2012/217037

[CR60] Sharma P, Kumar A, Bhardwaj R (2016) Plant steroidal hormone epibrassinolide regulate- heavy metal stress tolerance in *Oryza sativa* L. by modulating antioxidant defense expression. Environ Exp Bot 122:1–9.

[CR63] Shubhangi K, Kirti S, Sofiya M, Suchita G (2017) Quantitative estimation of total phenolics and flavonoids in *Soymida febrifuga* leaves. Am J Phytomed Clin Ther 5(3):20

[CR64] Sumanta N, Haque CI, Nishika J, Suprakash R (2014) Spectrophotometric analysis of chlorophylls and carotenoids from commonly grown fern species by using various extracting solvents. Res J Chem Sci 4(9):63–69

[CR65] Surgun Y, Çöl B, Bürün B (2016) Differential expression analysis of boron transporters and some stress-related genes in response to 24-epibrassinolide and boron by semi-quantitative RT-PCR in *Arabidopsis thaliana* (L.) Heynh. Genetika 48(2):547–563. 10.2298/GENSR1602547S

[CR66] Surgun-Acar Y, Zemheri-Navruz F (2019) 24-Epibrassinolide promotes arsenic tolerance in *Arabidopsis thaliana* L. by altering stress responses at biochemical and molecular level. J Plant Physiol 238:12–19. 10.1016/j.jplph.2019.05.002 31121523 10.1016/j.jplph.2019.05.002

[CR67] Surgun-Acar Y, Zemheri-Navruz F (2022) Exogenous application of 24-epibrassinolide improves manganese tolerance in *Arabidopsis thaliana* L. via the modulation of antioxidant system. J Plant Growth Regul 41(2):546–557. 10.1007/s00344-021-10320-7

[CR68] Sytar O, Kumari P, Yadav S, Brestic M, Rastogi A (2019) Phytohormone Priming: Regulator for Heavy Metal Stress in Plants. J Plant Growth Regul 38:739–752. 10.1007/s00344-018-9886-8

[CR69] Tadjouri H, Medjedded H, Nemmiche S, Chadli R, Moulay M (2022) Stress response induced by cadmium in soybeans (*Glycine max* L.) and health risk assessment. Plant Physiol Rep 27(2):321–328. 10.1007/s40502-022-00663-y

[CR70] Tantray AY, Bashir SS, Ahmad A (2020) Low nitrogen stress regulates chlorophyll fluorescence in coordination with photosynthesis and Rubisco efficiency of rice. Physiol Mol Biol Plants 26(1):83–94. 10.1007/s12298-019-00721-0 32158122 10.1007/s12298-019-00721-0 PMC7036394

[CR71] Vardhini BV, Anuradha S, Sujatha E, Rao SSR (2010) Role of brassinosteroids in alleviating various abiotic and biotic stresses-a review. Plant Stress 4(1):55–61

[CR72] Velikova V, Yordanov I, Edreva A (2000) Oxidative stress and some antioxidant systems in acid rain-treated bean plants: protective role of exogenous polyamines. Plant Sci 151:59–66. 10.1016/S0168-9452(99)00197-1

[CR73] Wang B, Fang R, Zhang G, Liu N, Feng Z, Bu Y, Gong Y (2025) A CCT protein GmCIC5 activates *GmP5CS* to regulate proline accumulation and cadmium tolerance in vegetable soybean. J Hazard Mat 138319. 10.1016/j.jhazmat.2025.138319 10.1016/j.jhazmat.2025.138319 40262318

[CR74] Xue ZC, Gao HY, Zhang LT (2013) Effects of cadmium on growth, photosynthetic rate and chlorophyll content in leaves of soybean seedlings. Biol Plant 57(3):587–590. 10.1007/S10535-013-0318-0

[CR75] Yang R, Yang Z, Xing M, Jing Y, Zhang Y, Zhang K, Zhou Y, Zhao H, Qiao W, Sun J (2023) TaBZR1 Enhances Wheat Salt Tolerance via Promoting ABA Biosynthesis and ROS Scavenging. J Geneti Genom 50(11):861–871. 10.1016/j.jgg.2023.09.006 10.1016/j.jgg.2023.09.006 37734712

[CR76] Yaşar M, Sezgin M (2022) Investigation of genotype x environment interaction in second crop soybean cultivation. Acad J Agric 11(2):303–310. 10.29278/azd.1184355

[CR77] Yılmaz M (2024) Determination of important agricultural traits of some soybean (*Glycine max* (L.) Merr.) genotypes and adaptation in the Eastern Mediterranean Transition Zone. JOTAF 21(1):139–147. 10.33462/jotaf.1250402

