# Supplementary material for: 24-Epibrassinolide modulates antioxidant regulation and redox homeostasis in soybean exposed to cadmium stress
Source: Physiol Mol Biol Plants. 2026 Apr 27;32(5):1151–63. doi: 10.1007/s12298-026-01754-y (PMC13216426; doi:10.1007/s12298-026-01754-y)
Supplement: Supplementary file 1 — Supplementary Material 1 [file 12298_2026_1754_MOESM1_ESM.docx]

**Supplementary material**

**for**

**24-Epibrassinolide Modulates Antioxidant Regulation and Redox Homeostasis in Soybean Exposed to Cadmium Stress**

**Table S1.** Accession numbers, primer sequences, optimal annealing temperatures, and references for antioxidant-related genes in *Glycine max*

| **Gene name** | **Accession number** | **Primer sequences (5´-3´)** | **Tm (ºC)** | **References** |
| --- | --- | --- | --- | --- |
| *CSD5* | NM_001255882.1 | F-AGGTGTGGCTATCATCATCGG  R-TCACTCTCCCTGTCACATGG | 58 | This study |
| *FSD3* | NM_001250972.2 | F-GGGGAAACACCACAAGACATAT  R-AAGCCCATCCTGAACCAAAT | 58 | Lu et al., 2020 |
| *MSD1* | NM_001248137.2 | F-CCCATAATCGGAGCTGGCAT  R-CATGATTTCGCCGCTGATGG | 58 | This study |
| *CAT1* | NM_001250627.1 | F-TCTCTTTTGGACGACGAGGC  R-AAGGGGGTCAAAGTCAAGCC | 60 | This study |
| *APX1* | L10292.1 | F-ATGCGCTCCTCTAATGCTCC  R-GTCAAGACCGTTGTTAGCGC | 60 | This study |
| *POD* | XM_006575142.4 | F-ACGTTTGGAAGAGCTCGGTG  R-TGATCAGGTGTGCTCAGGTC | 60 | This study |
| *Actin-6* | NM_001289231.2 | F-GCAAGTGGTCGTACAACTGG  R-AGACGAAGGATGGCATGTGG | 58 | This study |

**References**

Lu, W., Duanmu, H., Qiao, Y., Jin, X., Yu, Y., Yu, L., & Chen, C. (2020). Genome-wide identification and characterization of the soybean SOD family during alkaline stress. PeerJ 8:e8457 <https://doi.org/10.7717/peerj.8457>
